# Contribution of Extracellular Particles Isolated from *Morus* sp. (Mulberry) Fruit to Their Reported Protective Health Benefits: An In Vitro Study

**DOI:** 10.3390/ijms25116177

**Published:** 2024-06-04

**Authors:** Neve R. Garrett, Ryan C. Pink, Charlotte Lawson

**Affiliations:** 1 Department of Comparative Biomedical Sciences, Royal Veterinary College, London NW1 0TU, UK; ngarrett21@rvc.ac.uk; 2 Department of Biological and Medical Sciences, Oxford Brookes University, Oxford OX3 0BP, UK; rpink@brookes.ac.uk; 3 School of Pharmacy and Biomedical Sciences, University of Central Lancashire, Preston PR1 2HE, UK

**Keywords:** extracellular vesicles, mulberry, *Morus alba*, *Morus nigra*, inflammation, proliferation, oxidative stress

## Abstract

*Morus* sp. (mulberry) has a long tradition of use as a medicinal treatment, including for cardiovascular disease and type 2 diabetes, being shown to have antioxidant properties and to promote wound healing. Extracellular vesicles (EVs) are sub-micron, membrane-enclosed particles that were first identified in mammalian bodily fluids. EV-like particles have been described in plants (PDVs) and shown to have similar characteristics to mammalian EVs. We hypothesised that some of the health benefits previously attributed to the fruit of *Morus* sp. could be due to the release of PDVs. We isolated PDVs from *Morus nigra* and *Morus alba* via ultracentrifugation and incubated THP-1 monocytes, differentiated THP-1 macrophages, or HMEC-1 endothelial cells with pro-oxidant compounds DMNQ (THP-1) and glucose oxidase (HMEC-1) or lipopolysaccharide (LPS) in the presence of different fractions of mulberry EVs. Mulberry EVs augmented ROS production with DMNQ in THP-1 and caused the downregulation of ROS in HMEC-1. Mulberry EVs increased LPS-induced IL-1β secretion but reduced CCL2 and TGF-β secretion in THP-1 macrophages. In scratch wound assays, mulberry EVs inhibited HMEC-1 migration but increased proliferation in both low and high serum conditions, suggesting that they have opposing effects in these two important aspects of wound healing. One of the limitations of plant-derived therapeutics has been overcoming the low bioavailability of isolated compounds. We propose that PDVs could provide the link between physiological dose and therapeutic benefit by protecting plant active compounds in the GIT as well as potentially delivering genetic material or proteins that contribute to previously observed health benefits.

## 1. Introduction

*Morus* sp., commonly known as mulberry, is a genus of flowering deciduous trees found growing abundantly across temperate regions, both in the wild and under cultivation. There are 24 *Morus* species that have been described, with at least 100 subspecies and varieties that have adapted to a wide range of different environmental conditions. Commercial and private collections have been described across Asia, Southern Europe (though there are collections in Northern and Central Europe), the southern parts of North America, and parts of Africa [1]. The most common and widely cultivated species are *Morus alba* (white mulberry), *Morus nigra* (black mulberry), and *Morus rubra* (red mulberry), though berry colour does not necessarily reflect the species under cultivation [2,3]. Berries from the *M. nigra* and *M. rubra* species have higher acidity compared to *M. alba* [4]; thus, *M. alba* berries may have a sweeter taste and are more often used now for processed mulberry products [3]. Nonetheless, all common mulberry species have long been utilised for their health-giving properties and with the benefit of modern analytical techniques, these properties have been widely attributed to the high levels of flavonoids and anthocyanins that different parts of the plants have been found to contain [5,6]. 

Different parts of the mulberry plant have been used for many centuries in traditional medicines, including the leaves and the bark as well as fruit, being considered to have a number of health benefits, including anti-diabetic, antioxidant, and anti-inflammatory properties, and to be protective in cardiovascular disease [7], type two diabetes [8] and for cognitive function in neuro-degenerative conditions such as Alzheimer’s Disease [9]. Several studies have identified some of the compounds that could be responsible for these benefits [10]. Resveratrol, 3, 5, 4′-trihydroxystilbene has been identified in mulberry extract [11]. Resveratrol is a well-described compound that is produced by a number of different plant species in response to a variety of different stressors [12]. It has been shown to have positive benefits in vitro and in vivo, including antioxidant activity, which is likely to be due to changes in gene expression [13] and the promotion of genome stability [14]. There are several other classes of molecules that have been identified, including essential amino acids such as α-lipoic acid [15] and flavonoids, including morusin [16,17], which also contribute to the known antioxidant and anti-inflammatory properties of mulberry extracts. One key limiting factor of the previous studies is that they often require supra-physiological concentrations of purified factors in order to show an effect, due to the well-described low bioavailability of these compounds and inter-individual variability [18].

Extracellular vesicles (EVs) are sub-micron, membrane-enclosed particles that were first identified in the bodily fluids of several mammalian species [19]. They have been variously classified based on size, likely method of release from the cell surface, and some characteristic surface or internal markers into large EVs (LEVs 100–1000 nm, also called microparticles, microvesicles, ectosomes) and small EVs (sEVs < 150 nm, also called exosomes). LEVs are released via a calcium-regulated outward budding from the cell surface, whilst sEVs biogenesis is via an early endosomal pathway, forming multivesicular bodies which involve ESCRT-dependent and -independent pathways to deliver to the cell surface for release [20]. It is to be noted that there is a large size overlap between sEVs and LEVs and no definitive or exclusive marker for one or the other type of EV has been identified. The tetraspanins CD9, CD63, and CD81, which were initially believed to be limited to sEVs, have been identified in LEVs; both types of EVs can carry similar cargo and can have similar effects in recipient cells. For this reason, a recent position paper from the International Society for Extracellular Vesicles has recommended that EVs not be subdivided but considered a single population [21]. It may be argued that this approach could preclude subtle differences in the action of the two subsets when interrogating their potential function. There is also a third type of vesicle called Apoptotic Bodies. These are of a more heterogeneous size range up to 5000 nm and are released by cells undergoing apoptosis [20].

EV-like particles have now been shown to be released by a wide range of different eukaryotic and prokaryotic organisms [22]. The release of plant-derived vesicles (PDVs) has been described [6]. PDVs have been shown to have similar characteristics to mammalian EVs, including a limiting plasma membrane, the presence of tetraspanins on the surface; a cargo that can include proteins, DNA, mRNA, and microRNA; and other forms of non-coding genetic material. Like their mammalian counterparts, PDVs are believed to be important for intercellular communication, both within an individual organism and also for cross-species communication [23]. A well-known example is arbuscular mycorrhizal (AM) symbiosis. This is an ancient and highly conserved mutualism between plant and fungal symbionts whereby nutrient exchange and cross-kingdom signalling occurs between fungi in the subphylum *Glomeromycotina* and the cortical cells of roots of vascular plants including angiosperms, mosses, and gymnosperms. AM is believed to help plants capture nutrients and to have played a vital role in the colonisation of land by plants [24]. Likewise, PDVs from edible plants have been shown to stimulate anti-inflammatory and antioxidant gene expression in animal models [23].

For this study, we hypothesised that some of the health benefits previously attributed to the fruit of *Morus* sp., and in particular, to dark (DM) and white (WM) mulberry, could be due to the release of PDVs and subsequent uptake by mammalian cells. We, therefore, sought to isolate PDVs from the fruit of both species (mulberry EVs) and characterise their efficacy in a range of well-established in vitro assays with human monocyte/ macrophage and endothelial cell lines.

## 2. Results

### 2.1. Isolation of Vesicles from Mulberry Fruit

**Figure 1:**
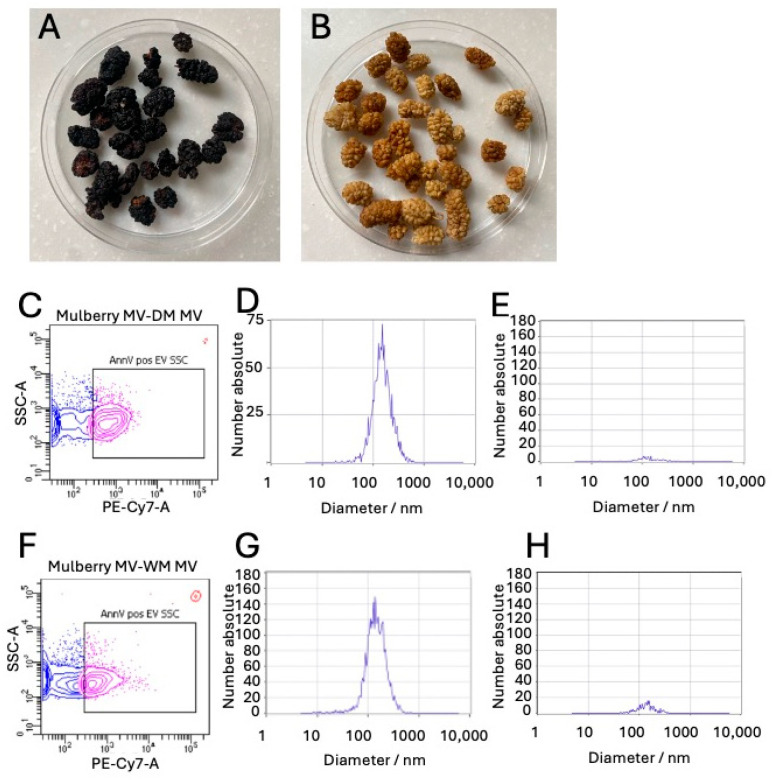
Identification of different PDV fractions from dark and white mulberry fruit. LEVs and sEVs were isolated sequentially from dried DM (**A**,**C**,**D**,**E**) and WM (**B**,**F**,**G**,**H**). Representative flow cytometry of LEVs (**C**,**F**), blue represents total LEVs, Pink represents Annexin V^+^ LEVs, red repreents enumeration beads, NTA of sEVs (**D**,**G**), and NTA of control fraction (**E**,**H**).

DM and WM were photographically assessed, and pH measured, to confirm that they were of different species (Supplemental data). Following the modification of our laboratory’s well-established ultracentrifugation protocol [25], we isolated EVs from both WM and DM. As shown in Figure 1, we were able to separate two populations: a population of LEVs which could be identified by the binding of annexin V to the surface and detection via flow cytometry (Figure 1C,F) and a population of sEVs which had a typical peak between 136 nm (WM sEVs) and 149 nm (DM sEVs; Figure 1D,G) per nanotracker analysis (NTA). In contrast, we could not detect EVs in the control samples (Figure 1E,H). We identified RNA and protein associated with the isolated EVs from both DM and WM (Table 1).

### 2.2. Modulation of Reactive Oxygen Species Production in Human Cells by Mulberry EV

**Figure 2:**
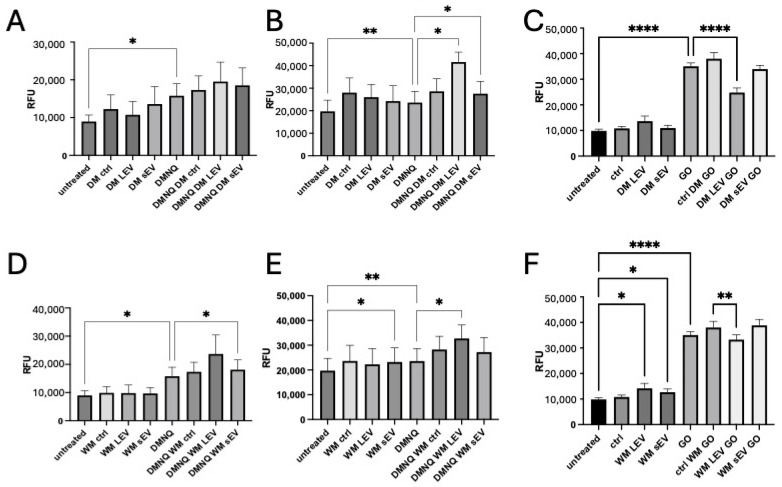
Mulberry EVs modulate ROS production in human cells in vitro. THP-1 monocytes (**A**,**D**) PMA-differentiated macrophages (**B**,**E**) and HMEC-1 (**C**,**F**) were pre-loaded with dihydrorhodamine-1,2,3 and treated with DMNQ (monocytes and macrophages) or GO (HMEC-1) in the presence or absence of DM EVs (**A**–**C**) or WM EVs (**D**–**F**). All treatments were in triplicate, n = 4 isolations of mulberry EVs. Repeated measures of one-way ANOVA followed by Fisher’s LSD. * *p* < 0.05, ** *p* < 0.01, and **** *p* < 0.0001.

In order to determine whether mulberry EVs could protect cells from pro-oxidant stimuli in vitro, we treated human THP-1 monocytes, PMA-differentiated macrophages, and HMEC-1 endothelial cells with appropriate stressors in the presence or absence of DM and WM EVs. DM EVs did not show any protective effect in monocytes (Figure 2A) and stimulated additional ROS production above DMNQ pro-oxidant stimulus in macrophages (Figure 2B), but in contrast, they significantly reduced the ROS production stimulated by GO in HMEC-1 endothelial cells (Figure 2C). WM EVs had a small but significant additive effect on ROS production in monocytes (Figure 2D) and macrophages (Figure 2E) and a reduction in HMEC-1 (Figure 2F).

### 2.3. Cytokine Secretion

**Figure 3:**
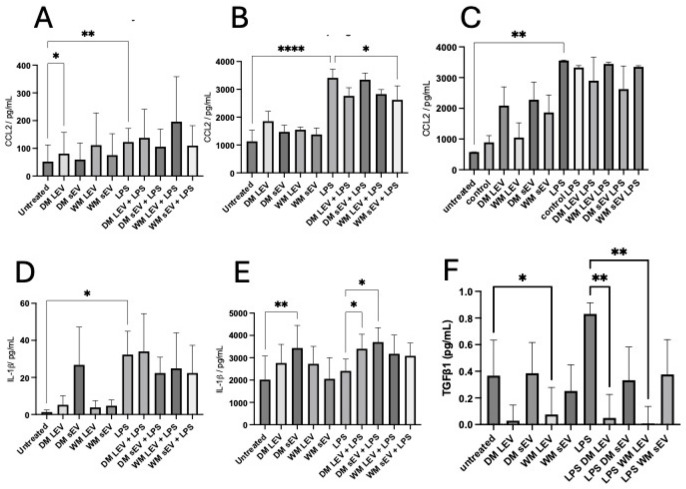
Mulberry EV modulation of LPS-stimulated cytokine secretion from THP-1 and HMEC-1. THP-1 monocytes (**A**,**D**), PMA-stimulated macrophages (**B**,**E**), and HMEC-1 (**C**) were treated with LPS in the presence or absence of mulberry EVs. The supernatants were assayed for CCL2 (**A**–**C**), IL-1β (**D**,**E**), and TGFβ1 (**F**). All treatments were measured in triplicate, n = 4 isolations of mulberry EV. Repeated measures of one-way ANOVA followed by Fisher’s LSD. * *p* < 0.05, ** *p* < 0.01, and **** *p* < 0.0001.

THP-1 monocytes, PMA-differentiated macrophages, and HMEC-1 endothelial cells were treated with LPS in the presence or absence of DM EVs and WM EVs. There was no effect of mulberry EVs on CCL2 secretion from THP-1 monocytes or HMEC-1 (Figure 3A,C); however, in THP-1 macrophages, there was a significant reduction in the LPS-induced secretion of CCL2 in the presence of WM sEVs (Figure 3B). We did not detect IL-1β or TGF-β1 from either quiescent or LPS-treated HMEC-1. IL-1β was secreted from THP-1 monocytes (Figure 3D) but was not modulated via any mulberry EVs. LPS-induced IL-1β secretion from THP-1 macrophages was significantly enhanced when they were co-treated with DM EVs (Figure 3E). No TGF-β1 was secreted from THP-1 monocytes. The secretion of TGFβ-1 was low in THP-1 macrophages, was increased after treatment with LPS, and was significantly decreased when co-incubated with mulberry LEVs (Figure 3F).

### 2.4. Mulberry EVs Have Opposite Effects on HMEC-1 Migration and Proliferation

**Figure 4:**
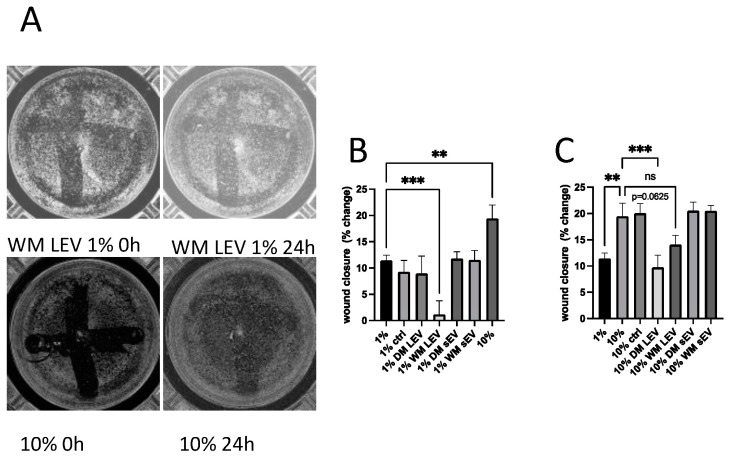
Migration of HMEC-1 endothelial cells is inhibited by mulberry EVs in an in vitro scratch (wound healing) assay. HMEC-1 were seeded onto 24-well plates at a sufficient density to form a confluent monolayer. After performing a “scratch”, whole wells were immediately imaged using a Tecan Spark Cyto imaging plate reader, [2× magnification], and again after 18 h, culture in either 1% or 10% EV-depleted FBS, in the presence or absence of mulberry EVs. (**A**) Representative images of scratches in 1% and 10% FBS at T0 and T = 18 h. (**B**) Averages of % change in confluency for wells incubated in 1% FBS. (**C**) Averages of % change in confluency for wells incubated in 10% FBS, n = 4. Repeated measures of one-way ANOVA followed by Fisher’s LSD. ** *p* < 0.01, and *** *p* < 0.001. n.s. not significant.

**Figure 5:**
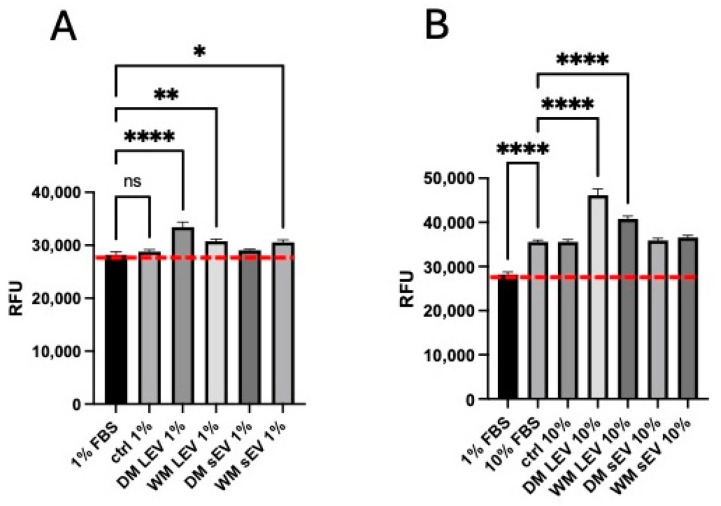
Proliferation of HMEC-1 is enhanced by mulberry EVs in low- (**A**) and high- (10%) FBS-containing medium (**B**). Cells were seeded at a low density in 96-well plates. The following morning, they were treated with 1% or 10% FBS in the presence or absence of mulberry EVs. Proliferation was measured after the addition of Alamar Blue. Red dotted lines depict level of proliferation in cells treated with 1% FBS only. One-way ANOVA followed by Fisher’s LSD; n = 4. * *p* < 0.05, ** *p* < 0.01, and **** *p* < 0.0001.

Extracts of mulberry fruit have been shown to be protective against vascular remodelling in animal models of hypertension [26] and to have antiangiogenic effects in human endothelial cells in vitro [27]. We, therefore, determined whether mulberry EVs could modulate both migration and proliferation of HMEC-1 endothelial cells. As shown in Figure 4, mulberry EVs, in particular the LEV fractions, were able to significantly reduce migration and wound closure after the scratching of a confluent endothelial cell monolayer. WM LEVs significantly inhibited closure when cells were grown in 1% FBS, and DM LEVs were effective in 10% FBS. Both DM LEVs and WM LEVs were able to significantly promote proliferation in the absence of an induced wound to the monolayer, in both 1% and 10% FBS (Figure 5). 

## 3. Discussion

*Morus* sp. (mulberry) has been used as a traditional medicine with a range of anti-inflammatory and antioxidant effects [17,28]. Several groups have extracted compounds that mirror these effects both in vitro and in animal models in vivo [26]. One common issue when considering the use of traditional therapies is the bioavailability of the active compounds. We, therefore, sought to determine whether some of the positive benefits of mulberry supplements could be delivered via naturally occurring EV-like particles (mulberry EVs). As shown in Figure 1 and Table 1, we were able to successfully isolate both small and large EV-like particles from dark and white mulberry fruit. We noted that approximately 10-fold more RNA was isolated from DM versus WM EVs. It has previously been demonstrated that low pH positively influences RNA extraction from EVs derived from mammalian cell lines [29], so the increased yield is linked to our finding that DM extracts were approximately 1 pH unit lower than WM extracts (Supplemental Data and [4]).

In order to determine whether mulberry EVs could protect cells from pro-oxidant stimuli in vitro, we treated human THP-1 monocytes, PMA-differentiated macrophages, and HMEC-1 endothelial cells with appropriate stressors (DMNQ or GO) in the presence or absence of DM and WM EVs. Overall, mulberry EVs had a stimulatory effect on ROS generation in both THP-1 monocytes and macrophages (Figure 2). In contrast, we observed a reduction in GO-induced ROS in HMEC-1 endothelial cells in the presence of mulberry EVs. In a previous study, mulberry polysaccharide extracts were shown to ameliorate oxidative stress in Hep2G liver cells in vitro [30]. This suggests that the mulberry fractions, cell type, pro-oxidant stimulus, or micro-environment is important for different effects.

Mulberry fruit is described as anti-inflammatory, with several reports demonstrating an effect of the reduction in cytokine secretion with ethanol extracts from mulberry [17]. We treated THP-1 monocytes, PMA-differentiated macrophages, and HMEC-1 endothelial cells with LPS in the presence or absence of mulberry EVs. Mulberry EVs did not influence CCL-2 production in THP-1 monocytes and HMEC-1 endothelial cells; however, there was a small but significant reduction in CCL-2 secretion in THP-1 macrophages when they were co-cultured with LPS and WM sEVs. There was no measurable secretion of IL-1β via HMEC-1. In THP-1 monocytes, IL-1β secretion was not influenced by mulberry EVs in the presence or absence of LPS but there was an overall stimulatory effect on IL-1β production in macrophages by DM EVs in the presence of LPS. There was no measurable production of TGF-β1 via THP-1 monocytes or HMEC-1 endothelial cells. In contrast, LPS was able to induce the secretion of TGF-β1 by THP-1 macrophages and this was inhibited via mulberry EVs (Figure 3). 

As shown in Figure 4 and Figure 5, mulberry EVs had contrasting effects on the migration and proliferation of HMEC-1. Migration was significantly reduced in the presence of mulberry EVs, whilst proliferation was significantly increased, both in low and high percentages of EV-depleted FBS.

Our findings suggest that mulberry EVs can separately influence the factors that regulate migration and proliferation responses, and it is intriguing to speculate whether this could be of benefit in individuals with vascular inflammatory disease. The finding that TGF-β1 release is downregulated by mulberry EVs, albeit only in THP-1 macrophages, in these experiments, lends further weight to this hypothesis. TGFβ has previously been described as being anti-proliferative for endothelium, with this effect being overcome through the addition of bFGF [31]. Furthermore, in that study, TGF-β induced an invasive (pro-migratory) response in 2d culture, which was inhibited via the addition of bFGF [31]. In an in vivo model of diabetic bone degeneration, the blockade of TGFβ using a neutralizing antibody led to a significant improvement in bone regeneration [32]. It is important to note that whilst the Alamar Blue proliferation assay (Figure 5) demonstrated a net increase in cell proliferation, we did not specifically measure apoptosis or necrosis. It would be interesting to follow this up to determine if the reduction in migration (Figure 4) is associated with cell death.

*Morus* sp. fruit has been suggested as an under-utilised natural source of health-promoting compounds [11], and extracts have been shown to stimulate a number of protective signalling pathways or inhibit cellular inflammation and invasion. Morusin and its derivatives are a group of flavonoids isolated from *Morus* sp. that have been shown to prevent the growth of a range of different cancer cell lines in vitro [16]. The studies have, furthermore, demonstrated that morusin acts on several different pathways, inhibiting NF-kB translocation [33] and suppressing STAT3 signalling pathways, leading to the reduced invasion of SK-Hep1 cancer cells [34]. *Morus* sp. extracts have been demonstrated to be protective against LPS-induced inflammatory cytokine secretion, reduce ROS production in mouse RAW-264 macrophages, and provide protection in an in vivo model of ulcerative colitis [17]. In the present study, when interrogating human macrophages, we found that ROS was increased and IL-1β secretion was enhanced in the presence of mulberry EVs, suggesting a tip towards M1 pro-inflammatory macrophages, whilst TGFβ1 was decreased. Further work. Including proteomics and HPLC is, therefore, required to gain a better understanding of whether the isolated mulberry EVs contain resveratrol, morusin, and other flavonoids at a physiologically relevant dose, or whether the measurable protein and miRNA content (Table 1) influences the subsequent cell behaviour. Metabolomics with EV-derived material is an emerging field with its own challenges due to the size of EVs. Likewise, investigating the uptake and metabolism of mulberry EVs in human cells could be beneficial for predicting pharmacokinetics and ADME properties in the future.

It is important to note that in many of the previous studies examining the potential health benefits of *Morus* sp., the concentrations of purified compound required to see an effect were not physiologically relevant, and if translated to a human clinical trial, would require that ingestion of high levels of dried or fresh fruit to give an effect. In the present study, the starting weight of dried fruit was 10 g for each isolation, which is well within the suggested limit of 30 g of any one type of fruit/day for health benefits, according to healthy eating guidelines [35]. 

There is increased interest in the potential for plant-derived EVs for the oral delivery of therapeutics, whether they be naturally occurring from edible plants or engineered to deliver specific drugs or compounds [36]. It is also worth considering that there could be sustainability benefits as well as health benefits since the extraction of PDVs from currently discarded parts of fruits after industrial food processing would mean less waste [37,38]. There is, therefore, a need to identify and understand the role of PDVs and how they contribute to the plethora of benefits previously reported in a range of different edible plants and traditional plant-derived medicinal products.

## 4. Materials and Methods

### 4.1. Materials

All general laboratory reagents and chemicals were from Sigma (Merck, Poole, Dorset, UK) unless otherwise stated. All tissue culture reagents and plastics were from Thermo Fisher LifeSciences (Loughborough, UK) unless otherwise stated.

### 4.2. Isolation of Mulberry EV

Dried fruits from dark mulberry (DM) were purchased online from Sussex Wholefoods (Sussex, UK), and white mulberry (WM) was purchased from The Refill Shop (St Neots, Cambridgeshire, UK). The species of each sample were photographically assessed by two UK mulberry fruit experts and additionally by testing the pH of reconstituted fruits (Supplemental data). For each isolation, 10 g was weighed out and re-hydrated in 10 mL 1×PBS in a 50 mL conical tube for 10 min on a roller (Thermo Fisher, Loughborough, UK) at room temperature. The fruit was then placed in a pestle and mortar, diced with a scalpel blade, and then pulped before returning to the 50 mL conical tube. The pulp was diluted to 30 mL with 1×PBS and centrifuged at 3000× *g* for 10 min at 4 °C in a serological centrifuge with a swing-out rotor. The resulting pellet was discarded, and the supernatant was filtered through a 40 μm cell strainer before recentrifugation at 3000× *g* for 10 min at 4 °C. The supernatant was decanted into a 33 mL thick walled polyallomer ultracentrifuge tube (Beckman Coulter, High Wycombe, UK) and centrifuged at 17,000× *g* for 30 min at 4 °C without braking in an XPN80 ultracentrifuge with a SW40Ti swing-out rotor (Beckman Coulter). The pellet (LEV) was resuspended in 1 mL 1×PBS and stored at −80 °C. The supernatant was carefully decanted into a clean ultracentrifuge tube and ultracentrifuged at 100,000× *g* for 90 min at 4 °C without braking. The supernatant was discarded, and the pellet (sEV) was resuspended in 12 mL 1×PBS and layered onto an 8 mL 30% sucrose “cushion” in a clean 33 mL ultracentrifuge tube. This was ultracentrifuged at 100,000× *g* for 60 min at 4 °C without braking. The cloudy layer at the interface was removed using a plastic “pastette” and diluted in 20 mL 1×PBS, then ultracentrifuged at 100,000× *g* for 60 min at 4 °C without braking. The top 1 mL of the supernatant was removed and stored as the control sample for each isolate. The pellet was resuspended in 1 mL 1×PBS and stored at −80 °C. n = 4 isolations were carried out for each fruit.

### 4.3. Nanotracker Analysis (NTA) of Mulberry EVs

A Zetaview PMX-120 (Particle Metrix, Analytik, Cambridge, UK) was used for NTA. A total of 1 μL of each sEV or control sample was diluted 1:1000 in 1 mL 1×PBS and applied to the machine according to the manufacturer’s instructions.

### 4.4. Flow Cytometry of Mulberry EVs

A FACS CANTO II (Becton Dickinson, Oxon, UK), calibrated daily using BD-CST beads according to the manufacturer’s recommendations, was used for the analysis of mulberry LEV. A total of 1 μL of each EV sample was diluted with 99 μL 1× Annexin V buffer and 2 μL Annexin-V-Cy7.7 (Thermo Fisher) and incubated on ice for 15 min, followed by further dilution to 500 μL with 1× Annexin V buffer. Gates were set up for total EVs, Annexin V EVs using the enumeration beads, and 1000 nm NIST Beads (Thermo Fisher). Ten-microlitre enumeration beads (Beckman Coulter) were added to each sample before immediate acquisition and analysis using BD FACSDiva Software v.9.0 (Becton Dickinson). The total and Annexin V-positive EV populations were calculated using the following equation:

$$n=\left[\#gated\;events\times 20\times dilution\;factor\right]÷[\#enumeration\;bead\;gated\;events]$$

### 4.5. Protein Assay of Mulberry EVs

EV samples were diluted 1 in 100 μL (sEV, control) or 1 in 1000 μL (LEV) RIPA buffer and subjected to sonication for 15 min at 30 °C. They were then assayed using a MicroBCA Assay (Thermo Fisher) according to the manufacturer’s instructions.

### 4.6. RNA Isolation of Mulberry EVs

Total RNA from each 250 μL sEV sample was isolated using the Qiagen Exo-RN-Easy kit according to the manufacturer’s instructions (Qiagen, Manchester, UK). For LEVs, a modified method was used—250 μL was centrifuged at 17,000× *g* in a benchtop microfuge. The resulting pellet was dissolved in Qiazol, and then, the remainder of the Exo-RN-Easy protocol was followed. RNA concentration was determined using a DeNovix DS-11 spectrophotometer (Cambridge Bioscience, Cambridge, UK).

### 4.7. Cell Culture

THP-1 human monocyte and HMEC-1 human endothelial cells were from ATCC and grown at 37 °C in 5% CO_2_. THP-1 cells were maintained in RPMI medium with 10% FCS and 50 μM of β-mercaptoethanol. For differentiation, THP-1 cells were incubated with 5 ng/mL of PMA for 72 h. For experiments, THP-1 cells were assayed in serum-free RPMI. HMEC-1 cells were maintained in MCDB-131 with 10 mM of L-glutamine, 1 μg/mL of hydrocortisone, and 10 ng/mL of human epidermal growth factor (hEGF; Biotechne, Abingdon, UK) and 10% FBS. For experiments, HMEC-1 cells were cultured in MCDB-131 without other additives but with L-glutamine and with exosome-depleted FBS, prepared using ultracentrifugation at 100,000× *g* for 18 h at 4 °C. This was used at 1% for ROS and LPS stimulation assays, and 1% or 10% for proliferation and scratch assays.

### 4.8. ROS Assays

As previously described in our laboratory [25,39], THP-1 monocytes were pelleted, washed, counted, and then incubated with 5 μM of dihydrorhodamine-1,2,3 (DHR) for 10 min in the dark. They were then seeded onto 96-well plates at 10,000 cells per well. EVs were added at 1:100 final dilution with or without the addition of 250 μM of 2,3-Dimethoxy-1,4-naphthoquinone (DMNQ; AbCAM, Cambridge, UK [40]). For THP-1 macrophages, they were seeded at 100,000 cells per well and differentiated with 5 ng/mL of PMA for 72 h. They were washed and incubated with DHR before treatment with EVs (1:100), with or without DMNQ. HMEC-1 cells were seeded at 10,000 cells per well and allowed to adhere overnight. The following morning monolayers were washed with 1×PBS and then incubated with MCDB-131, containing 1% exo-depleted FBS and 5 μM of DHR, for 10 min in the dark. EVs were added at a final dilution of 1:100 with or without 1 unit/mL of glucose oxidase as a stimulation for oxidative stress [41]. Plates were read on a Tecan Cyto Spark multimode plate reader (Tecan, Reading, UK). All treatments were in triplicate and n = 4 repeats were carried out.

### 4.9. Measurement of Cytokine Secretion

THP-1 monocytes were pelleted, washed, and plated into 24-well plates at 25,000 per well in serum-free RPMI. For macrophage experiments, 250,000 cells per well were plated in full-RPMI medium and cells were incubated for 72 h with 5 ng/mL of PMA, and then, monolayers were washed before the addition of serum-free RPMI. For HMEC-1 cells, 25,000 cells/well were seeded overnight onto 24-well plates. The following morning, monolayers were washed with 1×PBS followed by the addition of MCDB-131, containing 1% of EV-depleted FBS. For all cell lines, O55:B5 LPS was added at a final concentration of 1 μM with or without the addition of EVs at a 1:100 final dilution. Cells were incubated for 24 h before the removal of supernatant and centrifugation at 5000× *g* to remove any cell debris. Supernatants were stored at −80 °C and then assayed for CCL-2, IL-1β, and TGF-β, using Invitrogen Ready-Set-Go ELISAs according to the manufacturer’s instructions (Thermo Fisher).

### 4.10. Proliferation Assays

HMEC-1 cells were seeded at 2000 cells/well in 96-well plates. The following day, the medium was removed, and the monolayers were washed with 1×PBS and then replaced with MCDB-131 containing 1% or 10% EV-depleted FBS but no other additives. EVs were added at a final dilution of 1:100, and plates were incubated for 24 h at 37 °C. After this time, 10 μL of Alamar Blue (Invitrogen, Thermo Fisher) was added to each well and plates were incubated for a further 4 h at 37 °C. The plates were then read on a Tecan Spark multimode plate reader (Tecan, Reading, UK) at 560 nm Excitation and 590 nm Emission according to the manufacturer’s instructions. 

### 4.11. Scratch Wound Assays

HMEC-1 cells were seeded at 25,000 cells/well in 24-well plates. The following day, the medium was removed and the monolayers were washed with 1×PBS. The monolayer was “scratched” in a cross shape using the plunger from a 1 mL syringe. Monolayers were washed again with 1×PBS and MCDB-131 containing 1% or 10% EV-depleted FBS but no other additives were added. Wells were imaged using a Tecan Spark multimode plate reader (Tecan, Reading, UK) using the whole well imaging tool which gave a value for confluency. Plates were incubated overnight and re-imaged after 18 h. The percentage difference in confluency from individual wells was calculated.

### 4.12. Statistical Analysis

All statistics were carried out using Prism 10 (Graphpad, San Diego, CA, USA). One-way Repeated Measures ANOVA followed by Fisher’s LSD was used for all normally distributed data and the Friedman Repeated Measures test followed by Uncorrected Dunn’s was used for non-parametric data. All experiments were repeated with four separate isolates of mulberry EVs. *p* < 0.05 was considered statistically significant. * *p* < 0.05, ** *p* < 0.01, *** *p* < 0.001, and **** *p* < 0.0001 have been used to annotate significant differences in the figures.

## 5. Conclusions

We have isolated extracellular vesicles from the fruit of two species of *Morus* sp. and shown that they can modulate inflammatory and proliferative responses in human cell lines in vitro. This study adds to the growing body of evidence that plant-derived EVs may provide the link between physiological dose and therapeutic benefit by improving bioavailability and the uptake of active compounds, as well as potentially delivering genetic material or proteins that contribute to previously observed health benefits in plant-derived medications and supplements.

## Figures and Tables

**Table 1:** Characteristics of EV from dark and white mulberry fruit.

	RNA ng/μL ± s.d.	Protein μg/μL ± s.d.	FCM Total EV /μL ×10^6^ ± s.d.	FCM Annexin V^+^ EV/μL ×10^6^ ± s.d.	NTA Particles ×10^10^/mL ± s.d.	NTA Median Size nm ± s.d.
DM * LEV	225 ± 174	103 ± 45	1.0 ± 0.2	0.2 ± 0.1	n.d.	n.d.
DM sEV	57 ± 23	1.3 ± 0.7	n.d.	n.d.	7.93 ± 5	149.35 ± 5.6
WM LEV	25 ± 4	139 ± 52	0.68 ± 0.2	0.29 ± 0.2	n.d.	n.d.
WM sEV	82 ± 25	1.2 ± 0.2	n.d.	n.d.	14 ± 8	135.75 ± 6.4

* DM dark mulberry; WM white mulberry; LEV large EV; sEV small EV; s.d. standard deviation; n.d. not done; FCM flow cytometry; NTA nanotracker analysis; average values for n = 4 separate EV isolations.

## Data Availability

The data that support the findings of this study are available from the corresponding author upon reasonable request.

## Supplementary Materials

The following supporting information can be downloaded at https://www.mdpi.com/article/10.3390/ijms25116177/s1.
