# Supplementary material for: Contribution of Extracellular Particles Isolated from Morus sp. (Mulberry) Fruit to Their Reported Protective Health Benefits: An In Vitro Study
Source: Int J Mol Sci. 2024 Jun 4;25(11):6177. doi: 10.3390/ijms25116177 (PMC11173249; doi:10.3390/ijms25116177)
Supplement: Supplementary file 1 [file ijms-25-06177-s001.zip › ijms-3016851-supplementary.pdf]

# Contribution of Extracellular Particles Isolated from *Morus* sp. (Mulberry) Fruit to Their Reported Protective Health Benefits: An In Vitro Study

**Neve R. Garrett, Ryan C. Pink and Charlotte Lawson**

Supplementary Data

Verification of probable species of mulberry fruits used in this study was carried out by independent UK mulberry experts using photographic evidence.
Mark Travis (growingmulberry@gmail.com) and Nick Dunn (P. F. Matthews Ltd.) independently advised that based on photographs provided the samples could be separate species of *M. Nigra* and *M. Alba*, though for definitive identification of the variety more information about the tree stock would be required.
Additionally, the pH of each fruit sample was tested.
2g of each mulberry fruit sample was rehydrated with 5mL ddH20 for 5 min at room temperature. Fruit was pulped and allowed to settle. The pH of the resulting supernatant was tested using pH testing strips (VWR Dosatest Test Strips pH2.0 – pH9.0, VWR, Lutterworth, Leicestershire, UK). WM was found to have a pH of 5.5, whilst DM was found to have a pH of 4.5. This is in agreement with a previous study by Ercisli and Orhan (https://doi.org/10.1016/j.foodchem.2006.10.054).
